# Antibiotic use among patients with febrile illness in a low malaria endemicity setting in Uganda

**DOI:** 10.1186/1475-2875-10-377

**Published:** 2011-12-20

**Authors:** Vincent Batwala, Pascal Magnussen, Fred Nuwaha

**Affiliations:** 1 Department of Community Health, Mbarara University of Science & Technology P. O. Box 1410, Mbarara, Uganda; 2 Centre for Health Research and Development, Faculty of Life Sciences, Copenhagen University, Thorvaldsensvej 57, DK1871 Frederiksberg C, Denmark; 3 Disease Control and Environmental Health, Makerere University School of Public Health, P. O. Box 7072, Kampala, Uganda

**Keywords:** Antibiotic treatment, febrile patients, malaria diagnosis

## Abstract

**Background:**

Uganda embraced the World Health Organization guidelines that recommend a universal 'test and treat' strategy for malaria, mainly by use of rapid diagnostic test (RDT) and microscopy. However, little is known how increased parasitological diagnosis for malaria influences antibiotic treatment among patients with febrile illness.

**Methods:**

Data collection was carried out within a feasibility trial of presumptive diagnosis of malaria (control) and two diagnostic interventions (microscopy or RDT) in a district of low transmission intensity. Five primary level health centres (HCs) were randomized to each diagnostic arm (diagnostic method in a defined group of patients). All 52,116 outpatients (presumptive 16,971; microscopy 17,508; and RDT 17,638) aged 5 months to ninety five years presenting with fever (by statement or measured) were included. Information from outpatients and laboratory registers was extracted weekly from March 2010 to July 2011. The proportion of patients who were prescribed antibiotics was calculated among those not tested for malaria, those who tested positive and in those who tested negative.

**Results:**

Seven thousand and forty (41.5%) patients in the presumptive arm were prescribed antibiotics. Of the patients not tested for malaria, 1,537 (23.9%) in microscopy arm and 810 (56.2%) in RDT arm were prescribed antibiotics. Among patients who tested positive for malaria, 845 (25.8%) were prescribed antibiotics in the RDT and 273(17.6%) in the microscopy arm. Among patients who tested negative for malaria, 7809 (61.4%) were prescribed antibiotics in the RDT and 3749 (39.3%) in the microscopy arm. Overall the prescription of antibiotics was more common for children less than five years of age 5,388 (63%) compared to those five years and above 16798 (38.6%).

**Conclusion:**

Prescription of antibiotics in patients with febrile illness is high. Testing positive for malaria reduces antibiotic treatment but testing negative for malaria increases use of antibiotics.

**Trial Registration:**

ClinicalTrials.gov: NCT00565071

## Background

Recent World Health Organization (WHO) guidelines recommend a universal 'test and treat' strategy for malaria, mainly by use of rapid diagnostic test (RDT) and microscopy in all transmission areas [1]. In rural settings, however, febrile outpatients present with multiple complaints at health facilities and receive antibiotics in addition to anti-malarial treatment. Although WHO recommends rational use of medicines, it is estimated that in developing countries, the proportion of patients treated according to clinical guidelines for common diseases in primary care is less than 40% in the public sector and 30% in the private sector [2].

Antibiotics are routinely prescribed for colds, non-specific upper respiratory tract infections and acute bronchitis [3,4], as concomitant medications to anti-malarials [5]. In almost all cases, these are viral, self-limited conditions in which antibiotic use does not enhance illness resolution and is not recommended [6,7]. For other infections, such as otitis media, antibiotics provide some benefit, but the value of their use as first-line treatment has been debated [8].

Even for conditions in which antibiotic use might be justified, experts have expressed concern about substantial overuse [4] that impacts on health care costs. Inappropriate use of these drugs promotes antimicrobial resistance [9], adverse drug reactions and erodes patient confidence in health services [2]. In absence of urgent and corrective actions, the world is heading towards a post-antibiotic era in which many common infections will no longer have a cure and, once again, kill unabated [10,11].

Although the Uganda national malaria guidelines now recommend confirmation of parasitaemia before initiation of treatment, data on how results of use of malaria diagnostics influence antibiotic treatment among febrile outpatients is lacking. The current study assessed antibiotic prescribing rates among febrile outpatients attending rural health centres (HCs) where feasibility of rolling out parasitological diagnosis for malaria was being tested. The primary outcome measures were the proportions of patients with febrile illness that were prescribed antibiotics when: not tested for malaria, they test positive and when they test negative.

## Methods

### Study design

Data collection on antibiotic treatment was carried out within a cluster randomized feasibility trial of presumptive malaria diagnosis (control) and two diagnostic interventions (microscopy and RDT). Fifteen out of twenty sub-county level government HCs in a district of low malaria endemicity in Uganda were randomly selected for the trial. HCs were the primary sampling units, and were allocated to the three diagnostic arms using simple randomization. Finally, there were five HCs per arm (diagnostic method in a defined group of patients).

### Setting

The trial was carried out in Bushenyi district in south-western Uganda. The district headquarter is located at about 320 km from the capital city Kampala. The district is mainly rural with a total land area of 3,949 sq. km. It is endowed with diverse natural resources that include arable land, forests, large lake water bodies (Lakes Edward, George and Kazinga Channel), Queen Elizabeth National Park and minerals. The main economic activities are semi-intensive agriculture (growing crops and rearing animals), fishing and trade. The district is multi-ethnic with varying customs and norms. The main inhabitants are Banyankore and Bakiga. The total population is estimated at 731,392, and with 20 public HCs at sub-county level. The population distribution and density varies with physical geography. It is concentrated in the low-lying plateau zones of Sheema, Igara and Ruhinda; and sparse in the hilly-rough and rugged terrain of Buhweju and Bunyaruguru. The climate is relatively wet. The mean annual temperature range is 12.5°-30°C. Most of the district receives 1500-2000 mm of rainfall annually. Although Bushenyi experiences low and unstable malaria transmission, people of all ages are at risk. It is epidemic-prone, with occasional malaria outbreaks occurring shortly after the rains. The annual entomological inoculation rate is not known, but it was reported to be < 10 infective bites per person per year in the neighbouring district of Kanungu [12]. The trial commenced before Bushenyi was partitioned. However, partitioning did not affect the status of the trial HCs and the delivery of health services by the end of data collection. Additional description of the study setting has been published previously [13].

### Study procedures

#### Training of staff

A total of 74 clinical and laboratory staff received a one-day refresher training on-site. The training and subsequent study procedures were a scale-up of activities performed during the assessment of the accuracy of these malaria diagnostic methods [14]. All staff members were trained in theory by re-orienting them to the malaria treatment policy. Staff in the control arm were only re-oriented to the current malaria treatment guidelines but testing of patients was not performed. In the microscopy arm, members were, in addition, trained in 1) finger prick for collection of blood, 2) thick/thin blood smear preparation, 3) staining smears, and 4) blood smear reading. In the RDT arm, the staff were in addition, trained in 1) finger prick for collection of blood and 2) preparation and reading of *Paracheck^®^*. The staff in HCs with microscopy or RDT were instructed to treat patients for malaria according to test results. Treatment with antibiotics followed national guidelines. HC outpatient registers were modified to record additional variables such as the presenting complaints, drugs dispensed and to indicate those prescribed but out-of stock. The trained staff were charged with training those that were off-duty on the day of training. However, additional clarification was provided during supervision. Supervision by the study team was carried out weekly during the first two months and monthly thereafter. The district laboratory focal persons provided the routine quality control procedures in both microscopy and RDT arms.

### Description of the diagnostic arms

#### Presumptive (control) arm

Patients presenting with fever (by statement or measured) were enrolled to receive service without parasitological confirmation of malaria. Patients were treated on the basis of signs and symptoms only.

#### Microscopy arm

All patients presenting with fever (by statement or measured) were enrolled. The laboratory assistants prepared thick and thin blood smears by finger-prick using sterile blood lancets on separate frosted slides. Standard staining was performed using the Field's stain method. Laboratory assistants were only familiar with this staining technique. Blood films were read at magnification X1,000. Each film was graded as positive (asexual malaria parasites seen) or negative (no malaria parasites seen) based on inspection of 200 fields. Microscopy test results were recorded in the laboratory registers.

### Rapid diagnostic test arm

Patients presenting with fever underwent rapid testing with the "*Paracheck^®^*" device (Orchid Biomedical Systems, Goa, India). *Paracheck Pf^® ^*is based on the detection of histidine rich protein-2 (Pf HRP-2) produced by *Plasmodium falciparum *trophozoites and young gametocytes. The specimens were drawn by trained nurses or laboratory assistants using a simple finger-prick. The test preparation and interpretation were done following manufacturer's instructions and standard operating procedures. The test was considered positive when the antigen line was visible in the test window and negative when only the control band was visible. RDT results were recorded in the outpatient registers.

### Data collection

All outpatients presenting at the study HCs with fever (by statement or measured) from March 2010 to July 2011 were enrolled. Data collection was carried out weekly by the research assistants by extracting information from the laboratory and outpatient registers.

### Statistical analysis

The collected data were manually checked and cleaned. Data were double entered by two trained database assistants in a customized entry template with in-built consistency checks in EpiData 3.1 software (The EpiData Association, Odense, Denmark). The two data sets were validated to check for entry errors. Before analysis in Stata version 10 (Stata Corp LP, College Station, Texas, USA), the data were declared a cluster design using the "svyset command" with HCs as primary sampling units. Further, the Poisson regression model was fitted while accounting for clustering. Probability values (*p*-values) were set at 0.05 and confidence intervals (CI) were calculated at the 95% level. Socio-demographic and symptom data were presented using descriptive statistics: distribution by age, number and percent of patients with positive and negative results, and those receiving drugs.

### Ethical approval

The study was approved by Makerere University School of Public Health Higher Degrees Research and Ethics Committee; and the Uganda National Council for Science and Technology (Ref: HS 209). The study was registered with the Clinicaltrials.gov (NCT00565071).

## Results

### Description of the study population

**Figure 1:**
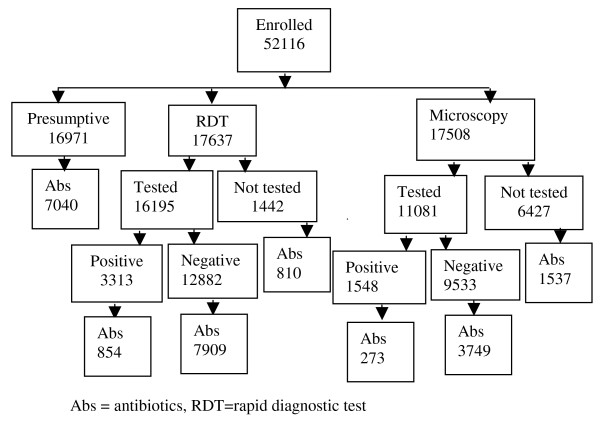
**Study profile**.

The study was carried out in 15 sub-county level government HCs located in an area of low malaria transmission intensity. Overall, 52,116 outpatients presenting with fever were enrolled: in the presumptive arm 16,971; microscopy arm 17,508; and RDT arm 17,637 (Figure 1). There were 8,552 children under five years (16.4%) with a median age of two [inter-quartile range one to three years]. The median age for those five years and above was 21 [inter-quartile range 13-34 years]. The overall age range was five months to ninety five years. The presenting symptoms of patients are presented in Table 1.

**Table 1:** Selected characteristics of study sample

Characteristic	Presumptive (%) [Int. range]	RDT (%) [Int. range]	Microscopy (%) [Int. range]
Level at the health delivery system	Sub-county	Sub-county	Sub-county
Number of health centres per arm	5	5	5
Total enrolment	16971	17637	17508
Gender (female)	10007(59.0)	10045(57.0)	10440(59.6)
Median age (in years)			
< 5 years	2 [1-3]	2 [1-3]	1.1 [1-3]
≥ 5 years	21 [13-34]	22 [14-35]	20 [13-34]
Non-specific URTI			
< 5 years	1554(52.9)	1626(59.9)	1350(46.6)
≥ 5 years	3635(25.9)	5432(36.4)	2643(18.1)
Otitis media			
< 5 years	6(0.2)	16(0.6)	20(0.7)
≥ 5 years	24(0.2)	8(0.1)	24(0.2)
Sore throat			
< 5 years	24(0.8)	8(0.3)	24(0.8)
≥ 5 years	20(0.1)	204(1.2)	136(0.9)

Int. range = inter-quartile range, RDT = rapid diagnostic test, URTI = upper respiratory tract infection

### Types of antibiotics prescribed

Oral co-trimoxazole was prescribed to 11,862 patients (51.0%) and amoxicillin capsules to 5,986 (25.8%). These two antibiotics were the most commonly prescribed. Metronidazole was prescribed to 3,950 patients (17.0%), but in combination with other antibiotics. Doxycycline, erythromycin and ciprofloxacin were also prescribed, but in smaller quantities. Other drugs such as analgesics and anti-helminthics were prescribed to 49,574 patients (95.2%) and 10,330 (19.8%) respectively.

### Antibiotic use in patients who did not receive a parasitological test for malaria

In the presumptive arm 7,040 patients (41.5%) were prescribed antibiotics. In the microscopy arm 6,427 (37%) did not receive a parasitological diagnosis and of these 1,537 (23.9%) were prescribed antibiotics. In the RDT arm, 1442 (8%) did not receive a parasitological diagnosis and of these 810 (56.2%) were prescribed antibiotics (Figure 1). Prescription of antibiotics was more common for children less than five years of age as compared to those who were older (Table 2). The pattern of antibiotic prescription varied widely across health units: presumptive arm (range 40.7% to 42.2%), microscopy arm (range 22.9% to 25.0%) and in RDT arm (range 53.6% to 58.7%). Overall 9387 (38%) of the patients who did not receive a parasitological diagnosis were prescribed antibiotics.

**Table 2:** Prescription of antibiotics in patients who did not receive parasitological diagnosis stratified by diagnostic method, age and health centre

Diagnostic method	< 5 years n(%)[95%CI]	≥ 5 years n(%)[95%CI]	Total n(%)[95%CI]
**Presumptive**			
Karungu	372(69.9) [66.0-73.8]	1327(39.0) [37.4-40.7]	1699(43.2) [41.6-44.7]
Kashenshero	*	1136(34.7) [33.1-36.3]	1136(34.7) [33.0-36.3]
Katunguru	376(59.5) [55.7-63.3]	1418(45.9) [44.1-47.6]	1794(48.2) [46.6-49.8]
Kyangyenyi	1000(56.6) [54.2-58.9]	238 (25.6) [22.8-28.4]	1238(45.9) [44.0-47.8]
Mutara	*	1173(35.2) [33.6-36.8]	1173(35.2) [33.5-36.8]
Total	1748(59.5) [57.8-61.3]	5292(37.7) [36.9-38.5]	7040(41.5) [40.7-42.2]
**RDT**			
Burere	20(76.9) [59.6-94.3]	148(56.1) [50.9-62.1]	168(57.9) [52.2-63.7]
Bushenyi	27(79.4) [65.1-93.7]	131(51.8) [45.6-58.0]	158(55.1) [49.3-60.8]
Katerera	20(76.9) [59.6-94.3]	123(51.0) [44.7-57.4]	143(53.6) [47.5-59.6]
Kyamuhumga	26(72.2) [56.9-87.6]	184(54.8) [49.4-60.1]	210(56.5) [51.4-61.5]
Kyeizoba	15(65.2) [44.2-86.3]	116(57.1) [50.3-64.0]	131(58.0) [51.5-64.4]
Total	108(74.5) [67.3-81.7]	702(54.1) [51.4-56.8]	810(56.2) [53.6-58.7]
**Microscopy**			
Bugongi	81(43.1) [35.9-50.2]	146(13.7) [11.7-15.8]	227(18.1) [16.0-20.3]
Kabira	123(47.3) [41.2-53.4]	208(18.3) [16.0-20.5]	331(23.7) [21.4-25.9]
Kabushaho	130(54.9) [48.5-61.2]	234(21.0) [18.6-23.4]	364(26.9) [24.6-29.3]
Kichwamba	123(54.7) [48.1-61.2]	216(23.8) [21.1-26.6]	339(30.0) [27.3-32.6]
Kigarama	107(48.2) [41.6-54.8]	169(15.8) [13.6-17.9]	276(21.3) [19.1-23.6]
Total	564(49.8) [46.9-52.7]	973(18.4) [17.3-19.4]	1537(23.9) [22.9-25.0]

CI = confidence interval, RDT = rapid diagnostic test, *children under-five records missing

### Antibiotic treatment in patients with a positive RDT or microscopy

Of the 3,313 patients who tested positive for malaria in the RDT arm, 854 (25.8%) were prescribed antibiotics. In the microscopy arm of the 1,548 with positive results, 273 (17.6%) were prescribed antibiotics (Table 3). Patients under five years were more often prescribed antibiotics than in those five years and above in both RDT and microscopy arms. Prescription of antibiotics also varied widely between health units (ranging from 15.7% to 27.3%).

**Table 3:** Prescription of antibiotics in patients with positive RDT or microscopy by age and health centre

Diagnostic method	< 5 years n(%)[95%CI]	≥ 5 years n(%)[95%CI]	Total n(%)[95%CI]
**RDT**			
Burere	50(42.7) [33.6-51.8]	104(19.4) [16.0-22.8]	154(23.6) [20.3-26.8]
Bushenyi	29(33.3) [23.2-43.4]	82(17.9) [14.4-21.5]	111(20.4) [17.0-23.8]
Katerera	47(45.2) [35.5-54.9]	115(21.3) [17.9-24.8]	162(25.2) [21.8-28.6]
Kyamuhumga	84(54.2) [46.3-62.1]	162(25.8) [22.3-29.2]	246(31.4) [28.1-34.6]
Kyeizoba	43(35.8) [27.1-44.5]	138(24.3) [20.7-27.8]	181(26.3) [23.0-29.6]
Total	253(43.4) [39.4-47.4]	601(22.0) [20.5-23.6]	854(25.8) [24.3-27.3]
**Microscopy**			
Bugongi	17(34.7) [20.9-48.5]	36(14.2) [9.9-18.6]	53(17.6) [13.2-21.9]
Kabira	23(37.7) [25.2-50.2]	38(12.8) [8.9-16.6]	61(17.0) [13.1-20.9]
Kabushaho	16(38.1) [22.8-53.4]	30(12.1) [8.0-16.2]	46(15.9) [11.6-20.1]
Kichwamba	26(41.9) [29.3-54.6]	39(13.8) [9.8-17.9]	65(18.9) [14.7-23.1]
Kigarama	23(41.8) [28.4-55.3]	25(12.6) [8.0-17.3]	48(19.0) [14.1-23.8]
Total	105(39.0) [33.2-44.9]	168(13.1) [11.3-15.0]	273(17.6) [15.7-19.5]

CI = confidence interval, RDT = rapid diagnostic test

### Antibiotic use in patients with negative RDT or microscopy

Overall, 11,658 patients (52.1%) with negative results were prescribed antibiotics (Table 4). Patients with negative RDT 7,909 (61.4%) received antibiotics prescription more often than those with negative microscopy results 3,749 (39.3%) [RR: 1.56; 95%CI: 1.41-1.73, *p *< 0.001]. Again children under five years of age with negative results in both RDT and microscopy arms were more often prescribed antibiotics than the older age group. Further, antibiotic prescription varied widely across health units (ranging from 38.4% to 62.4%).

**Table 4:** Prescription of antibiotics in patients with negative RDT or microscopy by age and health centre

Diagnostic method	< 5 years n(%)[95%CI]	All age ≥ 5 years n(%)[95%CI]	Total n(%)[95%CI]
**RDT**			
Burere	299(80.0) [75.6-83.8]	1258(58.5) [56.4-60.6]	1557(61.7) [59.8-63.6]
Bushenyi	180(81.1) [75.9-86.3]	767(54.0) [51.4-56.6]	947(57.7) [55.3-60.1]
Katerera	285(82.6) [78.6-86.6]	1190(63.0) [60.8-65.2]	1475(66.1) [64.1-68.0]
Kyamuhumga	485(81.1) [78.0-84.3]	1826(60.8) [59.0-62.5]	2311(64.2) [62.6-65.7]
Kyeizoba	353(79.0) [75.2-82.8]	1266(52.8) [50.8-54.7]	1619(56.9) [55.1-58.7]
Total	1602(80.6) [78.9-82.4]	6307(58.1) [57.1-60.0]	7909(61.6) [60.7-62.4]
**Microscopy**			
Bugongi	202(64.9) [59.6-70.3]	470(29.8) [27.5-32.0]	672(35.5) [33.4-37.7]
Kabira	213(65.5) [60.3-70.7]	543(32.7) [30.4-34.9]	756(38.0) [35.9-40.2]
Kabushaho	207(70.7) [65.4-75.9]	570(36.8) [34.4-39.2]	777(42.2) [39.9-44.5]
Kichwamba	189(70.3) [64.8-75.8]	572(39.4) [36.9-41.9]	761(44.2) [41.9-46.6]
Kigarama	195(64.9) [59.6-70.4]	590(33.0) [30.9-35.2]	785(37.6) [35.5-39.7]
Total	1006(67.2) [64.8-69.5]	2745(34.2) [33.2-35.2]	3751(39.4) [38.4-40.4]

CI = confidence interval, RDT = rapid diagnostic test

## Discussion

This article reports on a large assessment of the effect of malaria diagnostics on the probability of receiving antibiotics in a Ugandan population living in an area of unstable malaria transmission. The findings indicate that the rate of antibiotic treatment was high; there was a reduction in antibiotic prescription among patients with positive malaria test results; there was an increase in antibiotic treatment in those testing negative; the rate of antibiotic prescription was higher in children under five years of age; and antibiotic prescription varied widely across HCs suggesting that prescribers' behaviour is a big factor in use of antibiotics.

The level of antibiotic treatment reported here is higher than that demonstrated in Kabale [15], a district in the same region of Uganda with similar malaria endemicity, but comparable to that reported in Zanzibar [16]. In this study it is unlikely that symptoms alone justify this rate of antibiotic prescription as less than 5% of the patients would probably need antibiotics based on the clinical presentation. Other reasons such as expectations of the patient [17], service provider (prescriber) behaviour [18] and the social interaction between the patient and prescriber [17,18] have been cited. It was reported that for a satisfactory outcome of the consultation process, the clinician provides technically correct care, but this corresponds with the patient's expectations in order to legitimize the illness [18]. Also an earlier study [19] demonstrated that patient preference can stimulate inappropriate antibiotic prescribing. Further, other studies provided information about the other reasons for unnecessary antibiotic use [20-22]. Of particular importance are the inadequate staffing and the varying levels of professional training of staff manning outpatient clinics reported in a previous publication [12]. Understaffing is impacted by the heavy patient load, creating a need to finish the queue at the earliest possible time. Some reports also indicated that overuse of medicines is a consequence of diagnostic uncertainty by service providers, inappropriate unethical promotion of medicines by pharmaceutical companies, overworked health staff with limited time to spend with patients, and unrestricted availability of medicines [2,21,23]. These results complement these observations as diagnostic uncertainty and prescribers' behaviour were important determinants of antibiotic use in this study.

Patients who were in the RDT arm were more likely than those in the microscopy arm to be prescribed antibiotics. This difference is unlikely to be attributed to use of RDT. Generally in the microscopy arm, antibiotic prescription was low among: those not tested, who tested positive and those who tested negative. The trend of antibiotic use, however, was similar in the two diagnostic arms (decreasing among patients who test positive and increasing among negative patients). A more likely explanation for this difference in rates of antibiotic use among the diagnostic arms is prescribers' behaviour [i.e. higher likelihood of service providers more likely to treat with antibiotics in the RDT arm].

The rate of antibiotic prescription generally decreased among patients with positive results. This might indicate clinicians' acceptance of malaria-positive results as the only likely cause of illness at that point and therefore restrained from prescribing concurrent medications. However, the proportion of parasitaemic patients prescribed antibiotics reported here is higher than that observed in other settings [24]. Enormous resources have been invested in improving the targeting of anti-malarials but the concern of concurrent or otherwise antibiotic treatment has not received equal attention. Although antibiotic prescription to febrile outpatients appears complex because of frequent presentation with multiple complaints, antibiotic treatment in parasitaemic patients may be an indicator of the likely inability to utilize the clinical guidelines. Antibiotic prescribing for cough or non-specific upper respiratory tract infections was reported to be neither cost-effective nor cough-effective [25]. Therefore, there is need to enhance the treatment decisions at the lower level of care since staff manning these units have varying levels of professional training.

In this study, the chance of prescribing antibiotics increased if a febrile patient tested negative for malaria. These data are similar to what has been reported elsewhere [24,26-28]. Thus it appears that there is a compensatory antibiotic prescription in patients with negative results. This scenario of antibiotic prescribing has the potential to erode the financial savings that could accrue from widespread implementation of the universal 'test and treat' strategy for malaria. Besides the universal test and treat strategy does not provide adequate guidance on treating patients who test negative for malaria. Therefore, there is need to develop and implement guidelines regarding antibiotic treatment in febrile patients who test negative for malaria.

In some settings, interventions that promote rational antibiotic use have been shown to be effective. These emphasize careful diagnosis especially of upper respiratory syndromes, deferral of antibiotic use, and a watch-and-wait approach (along with symptom relief) for which antibiotics are not immediately indicated [29], and a targeted educational intervention [30]. Unless the use of antibiotics is curtailed, there is a prospect of higher costs, increased morbidity, and higher rates of death from common bacterial infections [2].

## Conclusions

Prescription of antibiotics in patients with febrile illness is high. Testing for malaria reduces antibiotic treatment in patients with positive results but increases in those testing negative. Antibiotic use also depends on age and prescriber behaviour. It is essential that malaria diagnostics are rolled-out in all primary level health units and guidelines for antibiotic treatment especially among children developed and distributed. In addition, continuing professional education for prescribers should be enforced.

## Conflicts of interests

The authors declare that they have no competing interests.

## Authors' contributions

All authors conceived and designed the study; VB and FN collected, analysed, interpreted the data and drafted the manuscript; PM critically revised the manuscript. All authors read and approved the final manuscript

## References

[B1] World Health Organization Guidelines for the treatment of malaria 2010 2 WHO, Geneva 25473692

[B2] WHO Medicines: rational use of medicines Fact sheet N°338 http://www.who.int/mediacentre/factsheets/fs338/en/# (accessed October 2011)21879340

[B3] Gonzales R Steiner JF Sande MA Antibiotic prescribing for adults with colds, upper respiratory tract infections, and bronchitis by ambulatory care physicians JAMA 1997 278 901 904 10.1001/jama.1997.03550110039033 9302241

[B4] Steinman MA Landefeld CS Gonzales R Predictors of broad-spectrum antibiotic prescribing for acute respiratory tract infections in adult primary care JAMA 2003 289 719 725 10.1001/jama.289.6.719 12585950

[B5] Dodoo AN Fogg C Asiimwe A Nartey ET Kodua A Tenkorang O Ofori-Adjei D Pattern of drug utilization for treatment of uncomplicated malaria in urban Ghana following national treatment policy change to artemisinin-combination therapy Malar J 2009 8 2 10.1186/1475-2875-8-2 19123926 PMC2647941

[B6] Stott NCH West RR Randomised controlled trial of antibiotics in patients with a cough and purulent sputum BMJ 1976 2 556 559 10.1136/bmj.2.6035.556 786428 PMC1688091

[B7] Gonzales R Bartlett JG Besser RE Cooper RJ Hickner JM Hoffman JR Sande MA Principles of appropriate antibiotic use for treatment of acute respiratory tract infections in adults: background, specific aims, and methods Ann Intern Med 2001 134 479 486 11255524 10.7326/0003-4819-134-6-200103200-00013

[B8] Takata GS Chan LS Shekelle P Morton SC Mason W Marcy SM Evidence assessment of management of acute otitis media, I: the role of antibiotics in treatment of uncomplicated acute otitis media Pediatrics 2001 108 239 247 10.1542/peds.108.2.239 11483783

[B9] Franco BE Altagracia Martínez M Sánchez Rodríguez MA Wertheimer AI The determinants of the antibiotic resistance process Infect Drug Resist 2009 2 1 11 21694883 PMC3108730

[B10] WHO World Health Day 2011. Urgent action necessary to safeguard drug treatmentshttp://www.who.int/mediacentre/news/releases/2011/whd_20110406/en/index.html (accessed October 2011)

[B11] WHO World Health Day 2011. Combat drug resistance: no action today means no cure tomorrowhttp://www.who.int/mediacentre/news/statements/2011/whd_20110407/en/index.html (accessed October 2011)

[B12] Okello PE Van Bortel W Byaruhanga AM Correwyn A Roelants P Talisuna A D'Alessandro U Coosemans M Variation in malaria transmission intensity in seven sites throughout Uganda Am J Trop Med Hyg 2006 75 219 225 16896122

[B13] Batwala V Magnussen P Nuwaha F Challenges to implementation of artemisinin combination therapy policy in Uganda Int Health 2010 2 262 268 10.1016/j.inhe.2010.07.002 24037867

[B14] Batwala V Magnussen P Nuwaha F Are rapid diagnostic tests more accurate in diagnosis of *Plasmodium falciparum *malaria compared to microscopy at rural health centres? Malar J 2010 9 349 10.1186/1475-2875-9-349 21126328 PMC3002380

[B15] Ndyomugyenyi R Magnussen P Clarke S Diagnosis and treatment of malaria in peripheral health facilities in Uganda: findings from an area of low transmission in south-western Uganda Malar J 2007 6 39 10.1186/1475-2875-6-39 17407555 PMC1851016

[B16] Msellem MI Mårtensson A Rotllant G Bhattarai A Strömberg J Kahigwa E Garcia M Petzold M Olumese P Ali A Björkman A Influence of rapid malaria diagnostic tests on treatment and health outcome in fever patients, Zanzibar: a crossover validation study PLoS Med 2009 6 e1000070 10.1371/journal.pmed.1000070 19399156 PMC2667629

[B17] Marinker M The doctor's role in prescribing J R Coll Gen Pract 1973 23 Suppl 2 26 30 4766228 PMC2635285

[B18] Chandler CI Mwangi R Mbakilwa H Olomi R Whitty CJ Reyburn H Malaria overdiagnosis: is patient pressure the problem? Health Policy Plan 2008 23 170 178 10.1093/heapol/czm046 18321889

[B19] Paredes P de la Peña M Flores-Guerra E Diaz J Trostle J Factors influencing physicians' prescribing behaviour in the treatment of childhood diarrhoea: knowledge may not be the clue Soc Sci Med 1996 42 1141 1153 10.1016/0277-9536(95)00387-8 8737432

[B20] Schwartz B Mainous AG Marcy SM Why do physicians prescribe antibiotics for children with upper respiratory tract infections? JAMA 1998 279 881 882 10.1001/jama.279.11.881 9516007

[B21] Hamm RM Hicks RJ Bemben DA Antibiotics and respiratory infections: are patients more satisfied when expectations are met? J Fam Prac 1996 43 56 62 8691181

[B22] Mainous AG Zoorob RJ Oler MJ Haynes DM Patient knowledge of upper respiratory infections: implications for antibiotic expectations and unnecessary utilization J Fam Pract 1997 45 75 83 9228917

[B23] Laxminarayan R Brown GM Economics of antibiotic resistance: a theory of optimal use J Environ Econ Manage 2001 42 183 206 10.1006/jeem.2000.1156

[B24] Reyburn H Mbakilwa H Mwangi R Mwerinde O Olomi R Drakeley C Whitty CJ Rapid diagnostic tests compared with malaria microscopy for guiding outpatient treatment of febrile illness in Tanzania: randomised trial BMJ 2007 334 403 10.1136/bmj.39073.496829.AE 17259188 PMC1804187

[B25] Hueston WJ Antibiotics: Neither cost-effective nor "cough" effective J Fam Pract 1997 44 261 265 9071245

[B26] Reyburn H Ruanda J Mwerinde O Drakeley C The contribution of microscopy to targeting antimalarial treatment in a low transmission area of Tanzania Malar J 2006 5 4 10.1186/1475-2875-5-4 16423307 PMC1360087

[B27] Bastiaens GJ Schaftenaar E Ndaro A Keuter M Bousema T Shekalaghe SA Malaria diagnostic testing and treatment practices in three different *Plasmodium falciparum *transmission settings in Tanzania: before and after a government policy change Malar J 2011 10 76 10.1186/1475-2875-10-76 21457570 PMC3080800

[B28] D'Acremont V Kahama-Maro J Swai N Mtasiwa D Genton B Lengeler C Reduction of anti-malarial consumption after rapid diagnostic tests implementation in Dar es Salaam: a before-after and cluster randomized controlled study Malar J 2011 10 107 10.1186/1475-2875-10-107 21529365 PMC3108934

[B29] Arnold SR Straus SE Interventions to improve antibiotic prescribing practices in ambulatory care Cochrane Database Syst Rev 2005 4 CD003539 16235325 10.1002/14651858.CD003539.pub2 PMC7003679

[B30] Razon Y Ashkenazi S Cohen A Hering E Amzel S Babilsky H Bahir A Gazala E Levy I Effect of educational intervention on antibiotic prescription practices for upper respiratory infections in children: a multicentre study Antimicrob Chemother 2005 56 937 40 10.1093/jac/dki339 16188917

